# Nourseothricin N-Acetyl Transferase: A Positive Selection Marker for Mammalian Cells

**DOI:** 10.1371/journal.pone.0068509

**Published:** 2013-07-04

**Authors:** Bose S. Kochupurakkal, J. Dirk Iglehart

**Affiliations:** 1 Department of Cancer Biology, Dana-Farber Cancer Institute; Boston, Massachusetts, United States of America; 2 Department of Surgery, Brigham and Women’s Hospital, Boston, Massachusetts, United States of America; Wake Forest Institute for Regenerative Medicine, United States of America

## Abstract

Development of Nourseothricin N-acetyl transferase (NAT) as a selection marker for mammalian cells is described. Mammalian cells are acutely susceptible to Nourseothricin, similar to the widely used drug Puromycin, and NAT allows for quick and robust selection of transfected/transduced cells in the presence of Nourseothricin. NAT is compatible with other selection markers puromycin, hygromycin, neomycin, blasticidin, and is a valuable addition to the repertoire of mammalian selection markers.

## Introduction

Initiatives like The Cancer Genome Atlas (TCGA) and Encyclopedia of DNA Elements (ENCODE) generate a wealth of “omics” data from normal and diseased tissue [1], [2]. The challenge is to use this data to decipher networks of interactions that drive phenotypes. Developing tools that enable experimental systems address complex hypotheses derived from “omics” data will have a broad impact. A plethora of tools like expression vectors and genome targeting constructs are readily available. However generating experimental models where multiple modifications have to be incorporated into the same cell is challenging. For example, elucidating underlying mechanisms driving transformation of primary cells or conversion of mature cells to stem cells require four or more genes and independent selection markers [3], [4]. Further, one additional selection marker is required when conditional expression is sought using two-plasmid systems.

Selection markers inactivate their cognate drugs that target cellular metabolism or are genotoxic. They are fundamental to genetic engineering cells and are used in a variety of expression and gene-targeting vectors. Vectors encoding markers for five drugs, Neomycin, Puromycin, Hygromycin and Blasticidin, all protein synthesis inhibitors and Zeocin or Phleomycin a DNA damaging agent are commercially available and widely used [5]. Among these, Zeocin/Phleomycin may induce genomic alterations even in cells harboring the selection marker [6], [7]. Therefore availability of additional selection markers that inactivate protein synthesis inhibitors will enable complex experimental design. This paper describes the adaptation of Nourseothricin N-acetyl transferase (NAT) as a selection marker for mammalian cells.

Norseothricin (NTC) is a member of the Streptothricin-class of antibiotics that inhibits protein synthesis by inducing miscoding. It is used as a selection marker for a wide range of organisms including bacteria, yeast, filamentous fungi and plant cells and is not known to have adverse side-effects on positively selected cells, a property cardinal to a selection drug. NTC is highly soluble in water (∼ 1 g/L) and stable in solution for 2 years at 4C [8]. NAT derived from *S. noursei,* is an enzyme that inactivates NTC by acetylating the beta-amino group of the beta-lysine residue of NTC [9]. Therefore, if NTC i) efficiently and rapidly kills naive mammalian cells, ii) is stable under cell culture conditions, iii) efficiently kills mammalian cells harboring resistance markers for Puromycin, Hygromycin, Blasticidin and Neomycin, and iv) if NAT expression efficiently rescues cyto-toxic effects of NTC, the NTC-NAT system would be a valuable addition to the repertoire of mammalian selection markers that inhibit protein synthesis.

## Materials and Methods

### Reagents

Human mammary epithelial cells immortalized with human telomerase (HMEC) was a gift from Jean Zhao [10]. BT549, MDA-MB468 and U2OS were obtained from ATCC. HEK293T and A2780 cells were obtained from Sigma-Aldrich. Phoenix cells were from Orbigen. The Nourseothricin acetyl transferase (NAT) ORF was a gift from Prof. Laura Knoll, University of Wisconsin. Puromycin, Neomycin, Blasticidin, Hygromycin were sourced from Sigma Aldrich and Nourseothricin (NTC) was obtained from Jena biosciences, Germany. Tissue culture media and reagents were obtained from Life Technologies. The retroviral Doxycycline regulated plasmids and Fetal Bovine Serum were purchased from Clontech. MTS reagent was sourced from Promega. The anti-HA antibody was obtained from Covance.

### Drug Sensitivity Assay

HEK293T, HMEC, BT549, MDA-MB468, U2OS and A2780 cells were plated in 96 well plates (5000 cells/well). After 12 hours, the plates were treated with NTC or Puromycin. Cell viability was measured using the MTS assay according to manufacturer’s protocol. Data was normalized to the MTS value obtained for untreated cells at Day 2. HMECs harboring resistance markers to Neomycin, Puromycin, Blasticidin and Hygromycin (H-NPBH) were plated in 96 well plates (5000 cells/well) and treated with NTC, Puromycin, Blasticidin or Neomycin 12 hours later. A separate plate was prepared for estimating the number of cells at the start of the experiment. Cell viability after 72 hours of exposure to drugs was measured using the MTS assay. The percentage of growth inhibition was estimated as described in the Developmental Therapeutics Program at NIH/NCI and plotted as a function of drug concentration [11].

### Generation of Retroviral Vectors

The codon usage of the NAT ORF was analyzed using JCat [12]. Unfavorable codons situated at the ends of the ORF were replaced with codons suggested by JCat and incorporated into the primers to generate the NAT ORF used for cloning (Fig. S1). The ORF for Puromycin acetyl transferase in the retroviral vector pRX-Tight Puro was replaced with the NAT ORF to generate pRXTN. The DNA fragment encoding p100 and p36 with a HA-tag at the N-terminus (HA-p100, HA-p36) were cloned into the multiple cloning site of pRXTN. The sequence of the vectors was confirmed by Sanger sequencing.

### Generation and Verification of Stable Cell Lines

Replication incompetent retroviruses encoding HA-p36 or HA-p100 under the control of a Tetracycline promoter and NAT were generated in Phoenix cells using standard protocols. H-N or H-PNHB cells were incubated with culture supernatant containing the viruses in the presence of 5 µg/ml Polybrene for 12 hours. Infected cells were treated with 50 µg/ml NTC for 2 weeks until resistant clones formed. To verify if H-N and H-NPBH cells expressed HA-p36 and HA-p100, pooled clones were plated in a six well plate and expression of p100-HA induced with 0.1/1 µg/ml Doxycycline (Dox) in the presence or absence of NTC. After 24 hours, whole cell lysates were prepared, protein amounts normalized using the Bradford assay and a Western Blot was performed using an anti-HA and anti-actin antibodies.

## Results and Discussion

**Figure 1:**
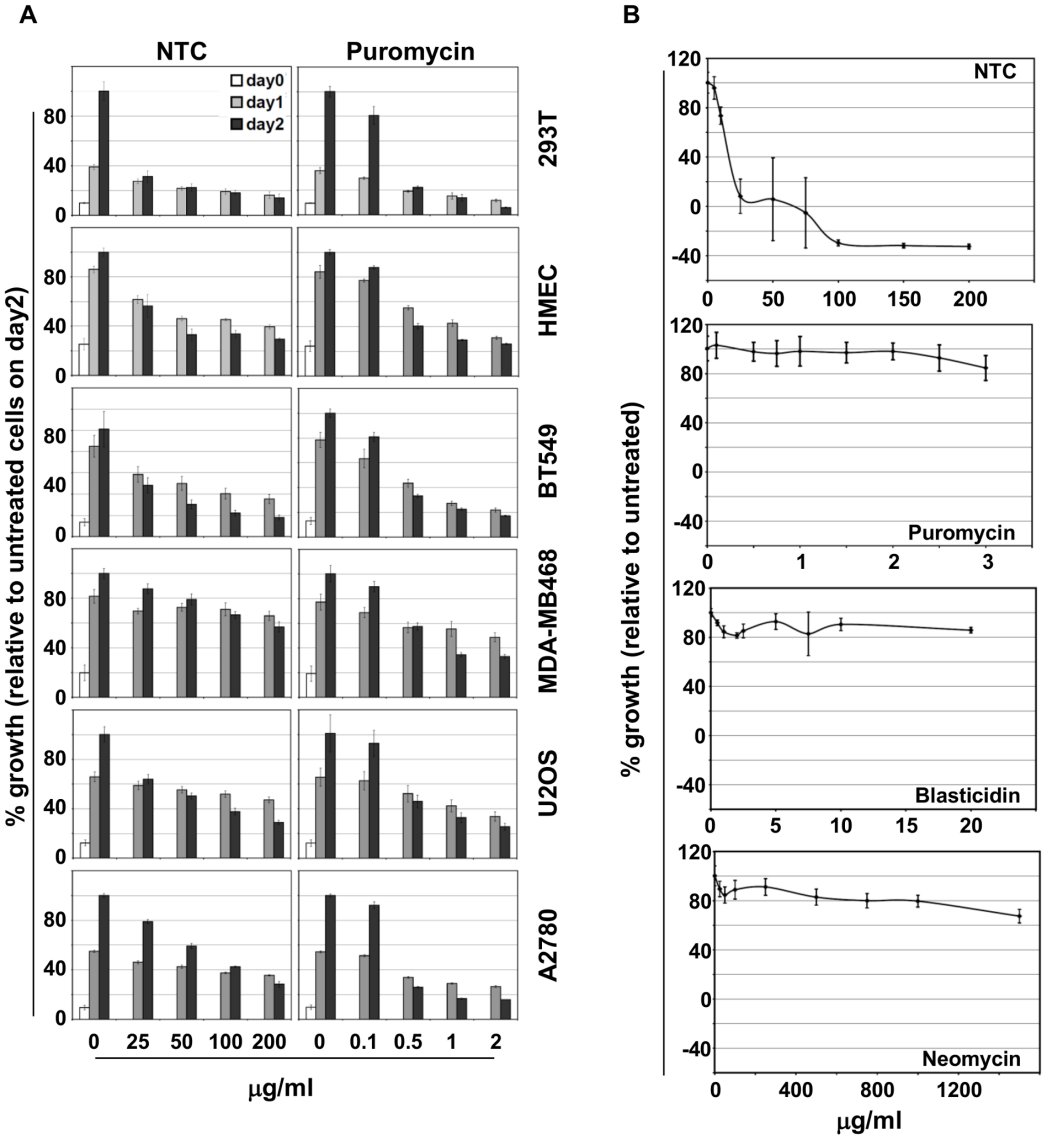
NTC efficiently kills mammalian cells and is compatible with other selection markers. (A) HEK293T, HMEC, BT549, MDA-MB-468, U2OS and A2780 cells were plated (5000 cells/well) in 96 well plates and treated with indicated drugs 12 hours post plating. Cell viability was measured using MTS on the day the drugs were added (day 0), and 24 (day 1) and 48 hours (day 2) later. The MTS values were plotted relative to cell growth devoid of drug on day 2. (B) HMEC cells harboring selection markers for Neomycin, Hygromycin, Blasticidin and Puromycin (H-NPBH cells) were plated in 96 well plates. After 12 hours, the cells were treated with the indicated concentration of drugs. Cell viability was measured 72 hours post treatment using MTS. The percentage of growth is plotted as a function of drug concentration.

First we compared cytotoxicity of NTC and Puromycin in a panel of six cell lines including HEK293T (Neomycin resistant; SV40 T antigen), Human Mammary Epithelial Cells (HMEC, hygromycin resistant; hTERT), BT549 (breast cancer), MDA-MB468 (breast cancer), U2OS (osteosarcoma) and A2780 (ovarian cancer). Although a higher dose of NTC compared to Puromycin was required to kill the cells, the toxicity profiles at the doses used are comparable (Fig. 1A). Susceptibility of the cells to killing with both drugs was time dependent and rapid. Therefore, similar to Puromycin, NTC kills naïve cells rapidly and can be used in typical transfection or infection based experiments or screens performed 48–60 hours post transfection/infection.

Although Puromycin, Blasticidin, Neomycin, Hygromycin and NTC inhibit protein synthesis, they bind unique ribosomal sites and inhibit different steps in the polypeptide synthesis process [13]. Moreover, enzymes that inactivate these drugs are highly specific and therefore expected to exhibit no cross resistance. We confirmed this hypothesis using a HMEC cell line harboring resistance markers to Puromycin, Neomycin, Hygromycin and Blasticidin (H-PNHB cells) to test the cytotoxicity of NTC in the presence of resistance markers to all these drugs. Hygromycin resistance was used to introduce hTERT in H-PNHB cells. Indeed, H-PNHB cells are acutely susceptible to NTC and 50 µg/ml NTC was sufficient to kill most cells within 72 hours (Fig. 1B). Taken together, results in Fig. 1 shows that NTC is a comparable selection drug for mammalian cells.

**Figure 2:**
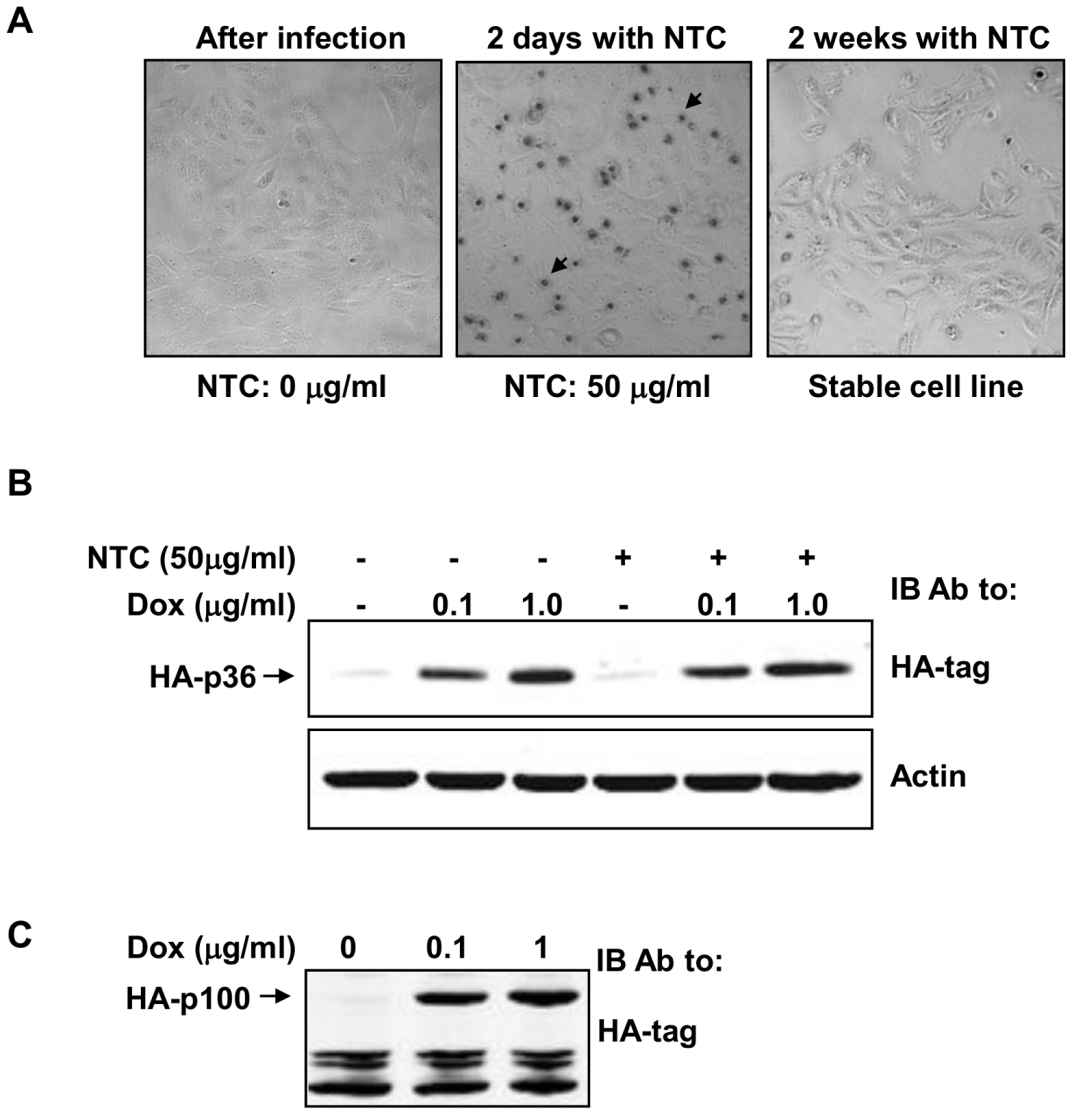
Expression of NAT allows generation of stable cell lines expressing heterologous proteins using NTC. (A) H-PNHB cells were infected with retroviruses encoding NAT and p100HA, and 12 hours later treated with NTC for selecting stable clones. Images of the infected cells, cells after 48 hours of selection and the generated stable cell line is shown. The black arrowheads indicate dying/dead cells. (B) Expression of HA-p36 in H-NH cells was induced for 24 hours with indicated amounts of Dox in the presence and absence of NTC. (C) Expression of HA-p100 in selected H-PNHB cells was induced with Doxycycline (Dox) for 24. Whole cell lystes were prepared after 24 hours and expression of HA-p100 was detected using an anti-HA antibody. Bands corresponding to HA-p36 and HA-p100 are indicated.

Encouraged by this observation, we generated a Tetracycline inducible retroviral plasmid harboring the humanized sequence of NAT (pRXTN; Fig. S1). DNA fragments encoding a 36 kDa (HA-p36) or 100 kDa (HA-p100) protein with a HA-tag at the N-terminus was cloned into this plasmid and retroviruses encoding the proteins were produced using Phoenix cells. H-NH (Hygromycin and Neomycin resistant HMEC) or H-PNHB cell line previously engineered to express the rtTA under Neomycin selection was transduced with the virus. The cells were split 24 hours post infection and cultured in the presence of 50 µg/ml NTC. Cell death (Fig. 2A, dark spots) was observed 2 days after addition of NTC. At 2 weeks proliferating colonies resistant to NTC with normal morphology were easily observed (Fig. 2A). To test if NTC has any residual effect on protein expression, we induced the expression of HA-p36 in H-NH-HA-p36 cells using 0.1 and 1.0 µg/ml Doxycycline in the presence and absence of 50 µg/ml NTC. We see clear bands of HA-p36 irrespective of the presence of NTC in the H-NH cells suggesting that NAT efficiently inactivated NTC (Fig. 2B). To further confirm NTC-NAT can be used to generate stable cell lines that are already resistant to Puromycin, Hygromycin, Blasticidin and Neomycin, we introduced HA-p100 in H-PNHB cells and selected stable clones using NTC. Doxycycline-induced expression of HA-p100 in the H-PNHB cells was confirmed by Western blot (Fig. 2C). Taken together our results indicate that NTC-NAT is an excellent selection system for mammalian cells. Together with NTC-NAT, it is now possible to easily generate mammalian cells with five stable modifications.

A variety of positive selection drug-marker combinations are available for modifying mammalian cells. Among them protein synthesis inhibitors, Puromycin, Hygromycin, Neomycin and Blasticidin are used widely because they do not require specialty media, they are efficient, have minimal confounding effects and can be used together at the same time. Identifying additional drug-marker pairs will enable complex experiments targeting multiple genes at the same time to validate findings from large-scale genome analyses like TCGA. Our results show that NTC-NAT is comparable to the Puromycin-Puromycin N-acetyl transferase system and meets the requirement for multiplexing gene targeting. Moreover, since genome-wide shRNA and ORF libraries are based on Puromycin and Blasticidin resistance markers, NTC-NAT can be used in conjunction with these publically available resources to introduce an alternate gene or shRNA [14], [15]. Since NTC kills cells devoid of NAT efficiently, it can be used in “transfection-analysis” experimental designs spanning 56–72 hours, typically used in genome-wide screens using shRNA or ORF libraries. High-level expression of target proteins are possible using bicistronic vectors encoding an IRES driven NAT [16]. Finally, the stability of NTC in culture medium and the broad toxicity of NTC across phyla makes it an extremely useful selection marker for combating contamination in industrial scale protein production in mammalian cells.

## Supporting Information

Figure S1 **Partially humanized sequence of NAT.** (A) Codon usage of the NAT sequence was analyzed using the web tool JCat (http://www.jcat.de/) with “Only partly optimization in order to apply site directed mutagenesis” option to generate a humanized sequence. The Codon Adaptation Index plots for the original sequence of NAT (NAT), codon optimized sequence (NAT_COS) and the changes incorporated in the final sequence used to construct the expression vector (NAT_Final) are shown. (B) Alignment of the three sequences NAT, NAT-COS and NAT_Final are shown.
(TIF)
