# Supplementary figures and images for: Nourseothricin N-Acetyl Transferase: A Positive Selection Marker for Mammalian Cells
Source: PLoS One. 2013 Jul 4;8(7):e68509. doi: 10.1371/journal.pone.0068509 (PMC3701686; doi:10.1371/journal.pone.0068509)

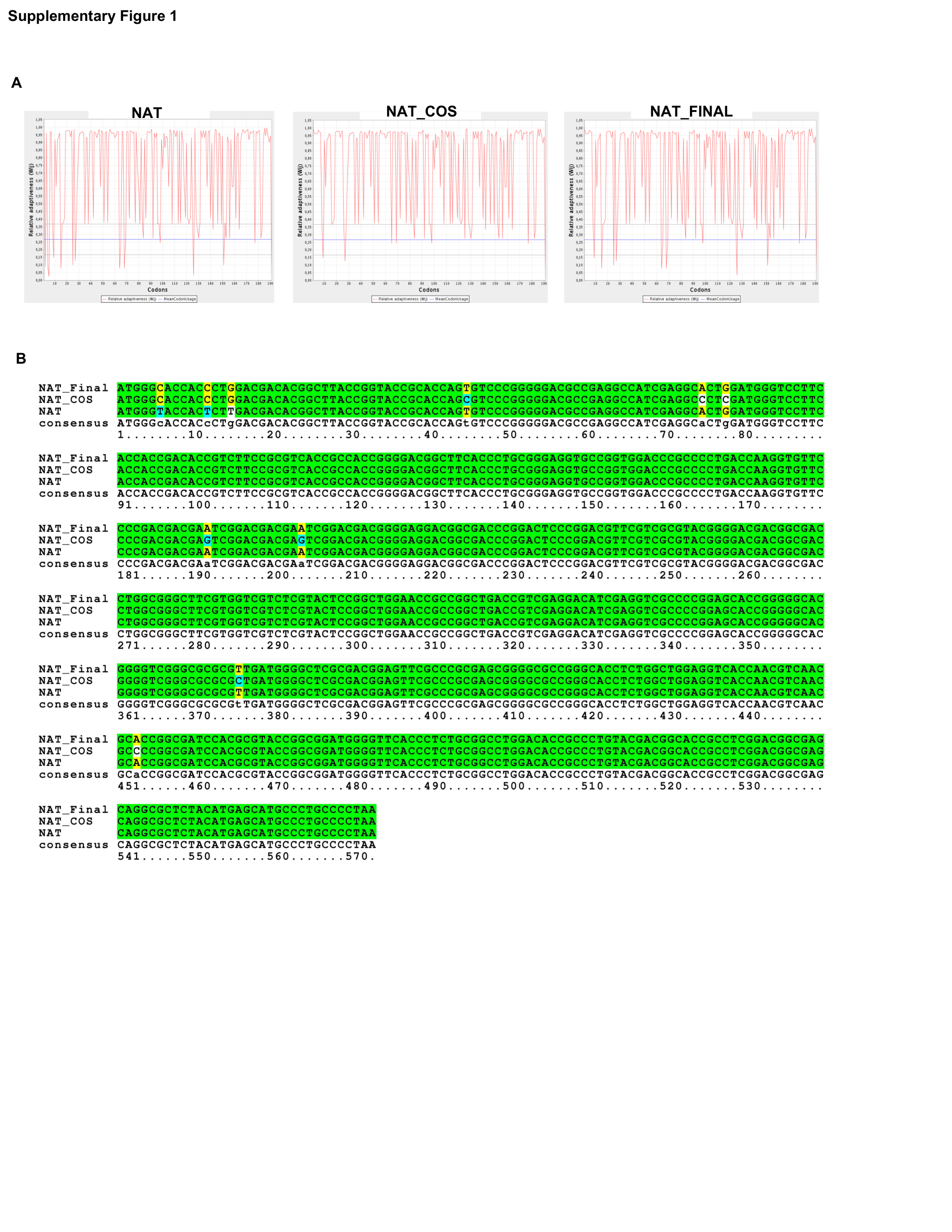

Supplement: Figure S1 — Partially humanized sequence of NAT. (A) Codon usage of the NAT sequence was analyzed using the web tool JCat (http://www.jcat.de/) with “Only partly optimization in order to apply site directed mutagenesis” option to generate a humanized sequence. The Codon Adaptation Index plots for the original sequence of NAT (NAT), codon optimized sequence (NAT_COS) and the changes incorporated in the final sequence used to construct the expression vector (NAT_Final) are shown. (B) Alignment of the three sequences NAT, NAT-COS and NAT_Final are shown. (TIF) [file pone.0068509.s001.tif]
